# Isolating DNA from sexual assault cases: a comparison of standard methods with a nuclease-based approach

**DOI:** 10.1186/2041-2223-3-25

**Published:** 2012-12-04

**Authors:** Alex M Garvin, Andrea Fischer, Jutta Schnee-Griese, Andrea Jelinski, Michel Bottinelli, Gianni Soldati, Monica Tubio, Vincent Castella, Nathalie Monney, Naseem Malik, Michelle Madrid

**Affiliations:** 1 Confarma France SARL, Zone Industrielle Canal d’Alsace, Hombourg, 68490, France; 2 Landeskriminalamt Baden-Württemberg, Crime Laboratory, Taubenheimstrasse 85, Stuttgart, 70372, Germany; 3 Molecular Diagnostics Laboratory, Genetic Forensic Unit, Via Tesserete 48, Lugano, CH, 6900, Switzerland; 4 Forensic Genetic Unit, University Center of Legal Medicine Lausanne and Geneva, Rue du Bugnon 21, Lausanne, CH, 1011, Switzerland; 5 University of Bern, Institute of Legal Medicine, Sulgenauweg 40, Bern, CH, 3007, Switzerland; 6 Los Angeles County Sheriff‘s Department, Forensic Biology, 1800 Paseo Rancho Castilla, Los Angeles, CA, 90032, USA

**Keywords:** Sexual assault, DNA purification, STR profiling, Differential lysis, Differex™, Erase Sperm Isolation Kit

## Abstract

**Background:**

Profiling sperm DNA present on vaginal swabs taken from rape victims often contributes to identifying and incarcerating rapists. Large amounts of the victim’s epithelial cells contaminate the sperm present on swabs, however, and complicate this process. The standard method for obtaining relatively pure sperm DNA from a vaginal swab is to digest the epithelial cells with Proteinase K in order to solubilize the victim’s DNA, and to then physically separate the soluble DNA from the intact sperm by pelleting the sperm, removing the victim’s fraction, and repeatedly washing the sperm pellet. An alternative approach that does not require washing steps is to digest with Proteinase K, pellet the sperm, remove the victim’s fraction, and then digest the residual victim’s DNA with a nuclease.

**Methods:**

The nuclease approach has been commercialized in a product, the Erase Sperm Isolation Kit (PTC Labs, Columbia, MO, USA), and five crime laboratories have tested it on semen-spiked female buccal swabs in a direct comparison with their standard methods. Comparisons have also been performed on timed post-coital vaginal swabs and evidence collected from sexual assault cases.

**Results:**

For the semen-spiked buccal swabs, Erase outperformed the standard methods in all five laboratories and in most cases was able to provide a clean male profile from buccal swabs spiked with only 1,500 sperm. The vaginal swabs taken after consensual sex and the evidence collected from rape victims showed a similar pattern of Erase providing superior profiles.

**Conclusions:**

In all samples tested, STR profiles of the male DNA fractions obtained with Erase were as good as or better than those obtained using the standard methods.

## Background

Although the rate of forcible rape continues to decline in the United States, it is still the third most common violent crime after aggravated assault and robbery, with 84,767 rape cases reported in 2010 [1]. Among the most useful types of forensic evidence from such cases is the vaginal swab taken from the victim, which often contains sperm, and thus sperm DNA, from the perpetrator. The goal of the crime laboratory processing such swabs is to obtain pure male DNA from the mixture of sperm from the assailant and epithelial cells from the victim in order to profile the male DNA with autosomal markers and to identify the perpetrator. Y chromosomal STR markers can be analyzed from a mixture of male and female DNA [2-6], but Y profiles do not determine the identity of the rapist because all males of the same paternal lineage have the same Y profile. Furthermore, Y chromosome profiles of unpurified DNA cannot be used when the victim is male.

The standard method for obtaining male DNA from a vaginal swab for autosomal profiling was first described by Gill and colleagues in 1985 [7] and is still being used in modified forms [8-10] by crime laboratories today. This method is commonly called differential lysis because the non-sperm cells are selectively lysed with detergent and Proteinase K, while the sperm are not lysed due to the heavily disulfide cross-linked proteins in the sperm head that resist protease treatment. The sperm are then pelleted by centrifugation, the supernatant containing the victim’s DNA is removed, and the pellet is washed multiple times to remove the remaining victim’s DNA prior to lysis of the sperm with a reducing agent to release soluble and relatively pure male DNA. The washing steps are tedious, difficult to automate, and result in sperm loss, and many attempts have been made to circumvent the need to wash the sperm pellet such as collecting the sperm by filtration [11,12], flow cytometry [13], and laser dissection [14-16], although none of these methods have been applied to routine casework.

An alternative to differential lysis that has been successfully applied to casework is Differex™ (Promega Corporation, Madison, WI, USA). Differex™ is designed to generate sperm DNA from a vaginal swab without having to wash the sperm pellet because the sperm are pelleted through an organic buffer having a density higher than the aqueous lysis buffer, which allows physical separation of the sperm pellet from the soluble residual DNA from the victim. Unfortunately, any of the victim’s DNA that remains in particulate form, either due to incomplete digestion of the victim’s cells or due to adsorption of the victim’s DNA to particles such as cotton fibers or sperm, will contaminate the male DNA fraction purified using Differex™. The Differex™ approach has been compared with differential lysis on mixtures of female epithelial cells and sperm [17], and was shown to be as efficient as a variant of differential lysis referred to as the two-step method [10]. In this study, no post-coital vaginal swabs were tested, and the most challenging mixture of female epithelial cells and sperm contained 10 female cells for each sperm, or a starting male-to-female DNA ratio of 1:20, which is much higher than that found in many post-coital vaginal swabs taken from rape victims. Many vaginal swabs from casework samples can have a starting ratio of male-to-female DNA of 1:100 or lower, especially when the victim waits many hours before reporting the crime and having the swab taken.

Another alternative to the differential lysis method has recently been described in which selective lysis of the non-sperm cells is performed using 0.1 N NaOH in the presence of the swab substrate, which is centrifuged in a spin basket to remove the victim’s DNA. The substrate is washed and treated with a nuclease to remove residual DNA from the victim and the sperm are then lysed with 1 N NaOH to release pure male DNA [18]. This method requires that the sperm firmly attach to the substrate and remain attached during the centrifugation and washing steps; no data have been published showing that this is the case with post-coital vaginal swabs, only with buccal swabs spiked with semen.

The approach of using a nuclease to remove residual DNA from the sperm pellet has previously been described [19]. In this study, the lysis buffer contained SDS detergent, which inhibits the nuclease (DNase I) and must be removed before adding the nuclease. Another approach to using a nuclease that does not require a buffer change involves using Triton X-100™ (Sigma Aldrich, Saint Louis, MO, USA), a non-ionic detergent that does not inhibit DNase I, in the lysis buffer instead of SDS [20]. A product using a nuclease to remove residual DNA from the sperm pellet without a buffer change has recently been commercialized by PTC Labs (Columbia, MO, USA) as the Erase Sperm Isolation Kit. The protocol for Erase is similar to the standard differential lysis protocol in many ways, and involves a Proteinase K/detergent lysis step to digest non-sperm cells and to elute the sperm from the swab substrate, centrifugation to pellet the sperm and removal of the supernatant containing the victim’s DNA, treatment of the sperm pellet with a nuclease to remove residual DNA from the victim (instead of washing the pellet), and simultaneous inhibition of the nuclease (by removal of the divalent cations needed for nuclease activity) and lysis of the sperm to generate soluble and pure DNA from the assailant. Since washing steps are eliminated, the protocol is faster and easier to perform than the standard differential lysis method. One should note that the female fraction from Erase will contain male DNA from non-sperm cells and can be used for Y-STR profiling.

Three of the laboratories in the current study (Bern, Lugano, and Lausanne) recently participated in a collaborative study where nine Swiss crime laboratories compared results of their standard methods for purifying male DNA from semen-spiked female buccal swabs [21]. The results of this work showed that intra-laboratory results were highly variable; and when buccal swabs spiked with 200 nl semen were processed, only two of the 18 profiles from the male fractions were male only, and the others were mixtures or ‘not interpretable’ (see Tables 1 and 2 in [21]). These results suggest that the methods now used by crime laboratories to process sexual assault cases can be improved. In order to determine whether Erase offers an improvement on the standard methods, the kit was tested by five crime laboratories in direct comparisons with their standard methods and the results of these comparisons are presented in this study.

**Table 1:** Summary of the methods used for this study

**Laboratory**	**Standard method for sexual assault cases**	**DNA purification**	**Quantitation method**	**STR profiling**
Los Angeles, CA, USA	Differential lysis	PCI/Micron YM100 (Millipore)	DUO (AB)	Identifiler Plus (AB)
Bern, Switzerland	Differential lysis	Qiamp/Micron YM100 (Qiagen/Millipore)	Quantifiler Human (AB)	NGM-Select (AB)
Stuttgart, Germany	Differential lysis	Charge-Switch (Invitrogen)	Quantifiler Human (AB)	NGM-Select or SEfiler (AB)
Lugano, Switzerland	Differex™	Qiamp (Qiagen)	Qubit (Invitrogen)	SGM-Plus (AB)
Lausanne, Switzerland	Differex™	Qiamp/Amicon Ultra 0.5 ml 30K (Qiagen/Millipore)	Quantifiler Human and Y Human Male (AB)	SGM-Plus (AB)

AB, Applied Biosystems (Carlsbad, CA, USA); Invitrogen (Carlsbad, CA, USA), Millipore (Billerica, MA, USA), Qiagen (Hilden, Germany).

**Table 2:** Quantitation of male DNA fractions

		**Total DNA in male fraction**^**a**^		**Percentage of male DNA**	
**Laboratory**	**Sperm input per swab cutting**	**Standard method**	**Erase**	**Standard method**	**Erase**
Los Angeles	50,000	7.7	3.5	99	68
	15,000	9.5	2.4	109	73
	5,000	0.6	0.3	32	193
	1,500	7.4	0.7	20	120
Bern	50,000	3.9	2.9	ND	ND
	15,000	0.5	2.2	ND	ND
	5,000	25.2	1.3	ND	ND
	1,500	0.1	0.7	ND	ND
Stuttgart	50,000	35.1	5.3	ND	ND
	15,000	11.4	4.5	ND	ND
	5,000	8.5	3.1	ND	ND
	1,500	4.2	0.0	ND	ND
Lugano	50,000	59.3	8.2	ND	ND
	15,000	38.4	10.7	ND	ND
	5,000	47.1	9.4	ND	ND
	1,500	67.2	5.1	ND	ND
Lausanne	50,000	21.7	72.0	100	59
	15,000	12.6	12.8	52	96
	5,000	12.3	6.0	26	66
	1,500	8.4	1.4	17	85

ND, not determined. ^a^Data presented as nanograms of DNA.

## Methods

### Semen-spiked buccal swab samples

Female buccal swabs (Cuticle Stick cotton swabs; Puritan Medical Products, Guilford, ME, USA) were obtained from a single healthy volunteer on five separate days, with four swabs taken per day, for a total of 20 female buccal swabs. Swabbing was done by rubbing each swab up and down four times with rotation on the inner surface of each cheek to assure even deposition of epithelial cells over the swab surface. The swabs were dried at room temperature in a ventilation hood for 24 hours before being placed in envelopes and stored at room temperature. Semen was provided by a single healthy volunteer. The sperm count of the semen was determined to be approximately 50,000 sperm/μl by hemocytometry. The semen was diluted to 10% and 1% in PBS immediately before use.

The buccal swabs were randomly assigned to one of five sets, one set per laboratory, with four swabs per set. Each swab was cut in half lengthwise, and to each of the two swab cuttings the same volume of semen solution was added. The first two swab cuttings of each set (labeled 1) were treated with 10 μl of 10% semen and received 50,000 sperm each. The second swab cuttings (labeled 2) were treated with 3 μl of 10% semen (15,000 sperm), the third swab cuttings (labeled 3) were treated with 10 μl of 1% semen (5,000 sperm), and the fourth swab cuttings (labeled 4) were treated with 3 μl of 1% semen (1,500 sperm). One swab half from each pair was randomly assigned to categories A and B, with four swab halves per category. The laboratories participating in the study received eight swab cuttings, labeled 1-4A and 1-4B. Each laboratory decided for itself which set (A or B) would be processed using their standard method or Erase to eliminate any possible bias.

### Timed post-coital swabs

Healthy volunteers (a single sexually active couple) provided vaginal swabs taken at 5 minutes, 6 hours, 24 hours, 34 hours, 48 hours, or 58 hours (six time points) after a single sex act. The crime laboratory in Lausanne processed the six vaginal swabs by cutting each swab in half and processing each half with either Differex™ or Erase. Buccal swab DNA from the volunteers was used to obtain reference profiles. This research was carried out on cell samples taken from humans and is in compliance with the Helsinki Declaration.

### Vaginal swabs from casework

The crime laboratory in Stuttgart chose evidence from four sexual assault cases (three vaginal swabs, one victim’s underwear with the appropriate controls; that is, buccal swabs from the victim and suspect when available). After analysis of the cases was completed using the established method, a portion of the remaining evidence was cut and processed using Erase.

### Methods for processing swab cuttings

The participating laboratories used the Erase Sperm Isolation Kit (PTC Labs) and followed the protocol included with the kit to process one set of swab cuttings. The other set of swab cuttings was processed by their standard method, as listed in Table 1. The standard methods were either versions of the selective lysis method first described by Gill and colleagues [7] or Differex™ (Promega).

### DNA purification, DNA quantitation, and STR profiling

The methods used by each participating laboratory for DNA purification, DNA quantitation, and STR profiling are given in Table 1 and in each case were the methods used by these laboratories to process routine casework and followed the manufacturer’s instructions. After the male and female DNA fractions were obtained with the two competing methods, all downstream steps of purification, quantitation, and STR profiling were identical for each sample pair in each laboratory. The Los Angeles laboratory used a mixture of phenol, chloroform, isoamyl alcohol, and 8-hydroxyquinalone (listed as PCI in Table 1) for DNA purification. A 1,020 ml sample of this reagent was made by mixing 500 ml phenol, 500 ml chloroform and 250 mg 8-hydroxyquinalone (all Sigma Aldrich, Saint Louis, MO, USA) with 20 ml isoamyl alcohol (Mallinckrodt Baker, Phillipsburg, NJ, USA). Only limited STR data (maximum of five loci plus amelogenin) are presented in this study to ensure the privacy of the donors.

## Results and discussion

### Mock sexual assault cases

Five sets of swab cuttings in duplicate containing female buccal cells and a known number of sperm (50,000, 15,000, 5,000, or 1,500) were sent to the five laboratories for processing. All 40 swab cuttings, processed either with the standard method or Erase, provided abundant amounts of DNA in the female fractions, and profiling of these fractions gave accurate female profiles in every case (data not shown). This is not surprising, as the swab cuttings each contained a large number of female epithelial cells (hundreds of thousands of cells, as is often the case with vaginal swabs) and these cells provide high nanogram to low microgram amounts of female DNA, which is far more than needed for profiling. Trace amounts of male signal were seen in the STR profiles of the female fractions using all methods, which can be accounted for by non-sperm cells in the semen, such as epithelial cells and macrophages, that are lysed by Proteinase K to release male DNA into the female fraction.

**Figure 1:**
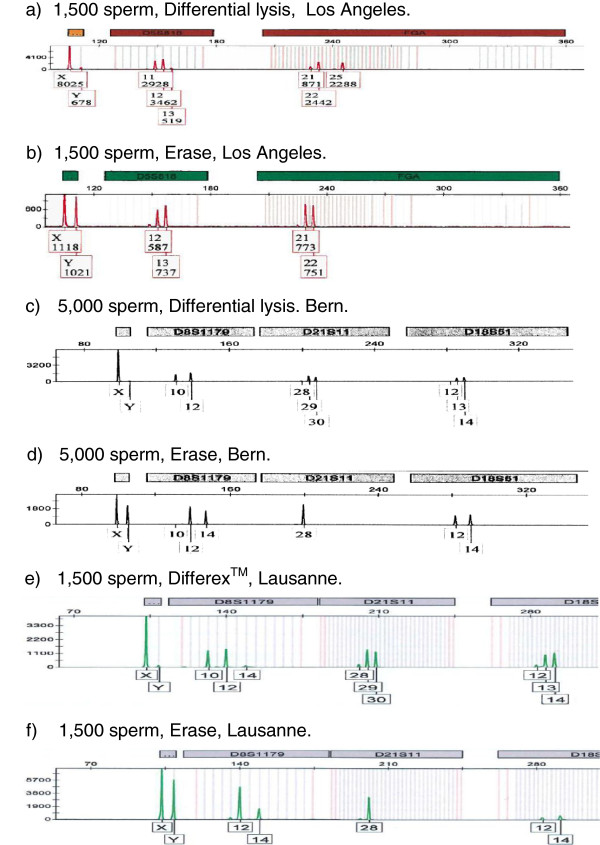
**Select STR profiles comparing standard methods (differential lysis or Differex™) with Erase.** The sperm input, method, and laboratory performing the analysis are noted. The correct male genotype is: D5S818 (*12*,*13*), FGA (*21*,*22*), D8S1179 (*12*,*14*), D21S11 (*28*), D18S51 (*12*,*14*). The correct female genotype is: D5S818 (*11*,*12*), FGA (*22*,*25*), D8S1179 (*10*,*12*), D21S11 (*29*,*30*), D18S51 (*13*,*14*). Differex™ (Promega Corporation, Madison, WI, USA). Erase Sperm Isolation Kit (PTC Labs, Columbia, MO, USA).

Table 2 presents the DNA yields for the male fractions of the 40 swab cuttings, and the percentage of male DNA in 16 of these samples from the two laboratories that routinely performed male-specific quantitation. The standard methods provided a higher DNA yield in 16 of the 20 direct comparisons (80%), although all 40 samples had enough DNA to obtain STR profiles. Erase provided male DNA of higher purity in five of the eight sets (62.5%) where purity was determined by quantifying male DNA as well as total DNA. In some samples where purity was measured, the results are clearly flawed; for example, three samples had a fraction of male DNA >100%, due to inaccuracies of the male and total DNA quantitation methods when low concentrations of DNA were being measured. In should be noted that such erroneous male DNA ratios are sometimes seen in casework. When comparing the purity data from Table 2 with the STR profiles in Figure 1, it is clear that the purity measured by quantitative PCR does not always correspond with the purity of the male fraction as determined by the peak area of contaminating female peaks in the STR profiles, and also does not always predict whether a profile will be obtained or not. Since the STR profiles of the male fraction are the most valuable data obtained from a vaginal swab, the quantitation results should be considered of secondary importance and male fractions should be profiled even when no male DNA is detected by quantitation.

Table 3 summarizes the genotypes obtained for male fraction DNA from the 40 swab cuttings. The correct male and female profiles were determined from buccal swabs obtained from the female and male contributors (data not shown). For the purposes of this study, a profile was considered full male when all loci were correctly identified as being of male origin and where the minor peaks represented less than 20% of the signal at each locus. The profile was considered a mixture when any of the loci had female signals of between 20% and 80% of the total signal at the locus, a partial male profile consisted of a male profile lacking one or more alleles at one or more loci, and the profile was considered female when the major alleles were female at all loci and represented more than 80% of the total peak area for each locus.

**Table 3:** Genotypes of the male fractions

	**Laboratory**				
**Sperm input**	**Los Angeles**	**Bern**	**Stuttgart**	**Lugano**	**Lausanne**
50,000 standard	Full male	Full male	Mixture	Mixture	Full male
50,000 Erase	Full male	Full male	Full male	Partial male (9/11)	Full male
15,000 standard	Full male	Mixture	Mixture	Mixture	Full male
15,000 Erase	Full male	Full male	Full male	Full male	Full male
5,000 standard	Mixture	Female	Mixture	Mixture	Mixture
5,000 Erase	Full male	Full male	Full male	Partial male (10/11)	Full male
1,500 standard	Mixture	Mixture	Mixture	Female	Mixture
1,500 Erase	Full male	Full male	Partial male (4/12)	Partial male (5/11)	Full male

For partial male profiles, the fraction of the loci that are correctly called is given in parentheses.

The results show conclusively that Erase provides superior profiles compared with the standard methods used by these laboratories. The standard methods gave full male profiles for five of the 20 samples (25%) while Erase gave full male profiles for 16 of the 20 samples (80%). None of the Erase profiles were mixtures or female profiles, although four of the 20 (20%) were partial male profiles, demonstrating that DNA prepared with Erase was essentially free of female DNA. As expected, the success of all three methods (differential lysis, Differex™, and Erase) was dependent on the number of sperm present on the swab cutting used as the starting material, such that cuttings with high numbers of sperm were more likely to give full male profiles. The most difficult samples, those with only 1,500 sperm, gave only female profiles or mixtures with the standard methods in all five laboratories, while identical samples processed with Erase gave full male profiles in three of the five laboratories and partial male profiles in the other two. A 1,500 sperm sample is the amount expected in 30 nl of a typical human semen sample having 50,000 sperm/μl, and represents 0.001% of the sperm present in 3 ml ejaculate with this sperm count. Such low numbers of sperm are often present on vaginal swabs taken from rape victims that wait many hours before reporting the crime and having the swab taken, and such low numbers of sperm are also sometimes found on the victim’ s clothing following sexual assault.

STR profiles of male DNA of three swab pairs processed in three different laboratories are presented in Figure 1. These are all swab cuttings with low numbers of sperm, either 1,500 (Figure 1a,b,e,f) or 5,000 (Figure 1c,d), where the performance difference between the methods is most pronounced. All panels contain the amelogenin locus because the X/Y peak ratio for amelogenin provides a convenient way to assess the quality of the male profile and the level of female contamination. The correct male and female alleles for the other loci are given in the figure legend. In all three cases, Erase provides full and correct male profiles, while the standard methods gave either a mixture (Lausanne and Los Angeles) or a female-only profile (Bern) for the loci shown.

### Timed post-coital swabs

**Figure 2:**
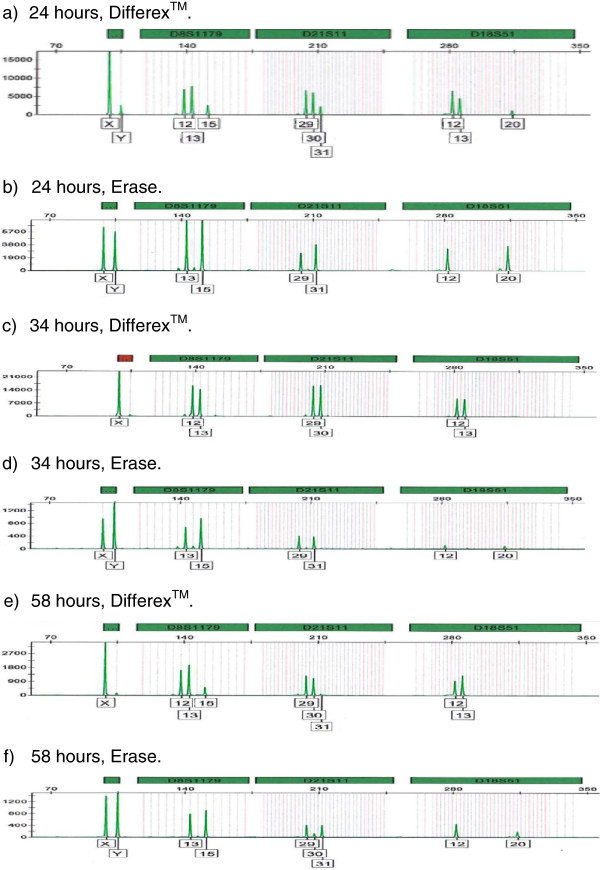
**Select STR profiles comparing Differex™ with Erase using timed post-coital swabs.** The times that the swabs were taken after sex are noted in the panels. The correct male genotype is: D8S1179 (*13*,*15*), D21S11 (*29*,*31*), D18S51 (*12*,*20*). The correct female genotype is: D8S1179 (*12*,*13*), D21S11 (*29*,*30*), D18S51 (*12*,*13*). Differex™ (Promega Corporation, Madison, WI, USA). Erase Sperm Isolation Kit (PTC Labs, Columbia, MO, USA).

Post-coital swabs taken at six different time points from 5 minutes to 58 hours after sex were provided by a single couple and processed by the laboratory in Lausanne using either Differex™ or Erase. For each time point, a single swab was cut in half and randomly assigned to either method. The early time points (5 minutes and 6 hours) gave full male profiles with both methods (data not shown), most probably due to the fact that early time points contain more sperm and are more likely to provide full male profiles. The later time points (24, 34, 48, and 58 hours) showed a marked difference between Differex™ and Erase, as documented in Figure 2. Swab cuttings processed with Erase gave full male profiles at each time point, including that taken at 58 hours (Figure 2f), while the profiles obtained with Differex™ were either mixtures (24 hours, Figure 2b) or female profiles (34 hours, Figure 2d; 48 hours, data not shown; and 58 hours, Figure 2e). The correct male and female alleles (as determined from buccal swabs from the donors) for the three loci are provided in the figure legend.

### Evidence samples from casework

**Figure 3:**
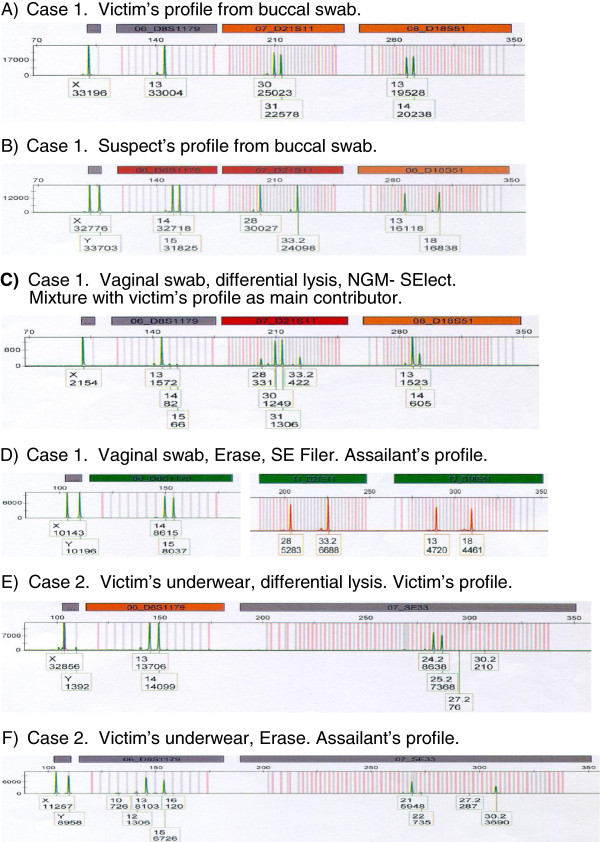
**Casework evidence from rape victims processed with the standard differential lysis or Erase.** Case 1: **(A)** victim’s profile obtained from a buccal swab, **(B)** suspect’s profile obtained from a buccal swab, **(C)** NGM SElect (Applied Biosystems, Carlsbad, CA, USA) profile of the sperm fraction DNA isolated using the standard method, and **(D)** SEfiler (Applied Biosystems) profile of the sperm fraction DNA isolated using Erase. Note that the loci in **(C)** and **(D) **are the same (amelogenin, D8S1179, D21S11, D18S51) but were obtained using different kits (NGM Select or SEfiler). Case 2: **(E)** profile of the sperm fraction DNA isolated using the standard method, and **(F)** profile of the sperm fraction DNA isolated using Erase. The correct female genotype for Case 2 as determined from a buccal swab is: D8S1179 (*13*,*14*), SE33 (*24*.*2*,*25*.*2*).

The laboratory in Stuttgart processed three vaginal swabs and one pair of a victim’s underwear taken from four different sexual assault cases using either conventional differential lysis or Erase as the method to obtain male DNA. In all four cases, better separation of the sperm fraction could be achieved using the Erase method. In two cases, both methods provided full male profiles with contributions of female DNA below 20% in the sperm fractions from the vaginal swab cuttings, with slightly fewer female contributions with the Erase samples (data not shown). In the other two cases, conventional differential lysis resulted in a mixture with a mostly female signal (Case 1, Figure 3c) or a female-only profile (Case 2, Figure 3e), whereas Erase provided full male profiles with both cases (Figure 3d,f). For Case 1, the victim’s and suspect’s profiles as obtained from buccal swabs are given for comparison (Figure 3a,b). By comparing Figure 3a,b,c,d, it is clear that the DNA profile from the sperm fraction of the vaginal swab prepared with the standard method matches that of the female victim (Figure 3c), while the male profile from the sperm fraction of the vaginal swab prepared with Erase matches that of the suspect (Figure 3d). Case 2 had no suspect, but the full male profile obtained with Erase (Figure 3f) could have been used to probe a database of STR profiles, while the profile obtained by differential lysis could not.

Some evidence samples from sexual assault will contain blood from the victim, and although the four casework samples tested in this study were not described as having large amounts of blood based on visual detection of blood, it has been shown that Erase works well on mixtures of female blood and semen (Christian Carson, PTC Labs, personal communication).

Although these cases had previously been closed and the full male profiles obtained with Erase were not instrumental in identifying and convicting the suspects, the improvement in quality of the male profiles obtained with Erase should result in higher identification and convictions rates for future cases. The crime laboratory in Stuttgart is therefore now using Erase routinely for casework.

## Conclusions

The differential lysis method describing a means to obtain relatively pure male DNA from a post-coital swab was published in 1985 [7] and is still in use today, 27 years later. In the cited publication, the authors washed the sperm pellet once to remove residual DNA from the victim (see legend to Figure 3, and Methods). Subsequent improvements to this method recommend a minimum of two wash steps [8]. These washing steps have the effect of physically removing the unwanted DNA from the victim but have the disadvantage of also removing some sperm and of being tedious and difficult to automate. Differex™, an alternative to differential lysis, is now used by numerous crime laboratories and is similar in many respects to differential lysis. In particular, the residual DNA from the victim is physically separated from the sperm by pelleting the sperm through an organic layer that the soluble DNA from the victim cannot enter, and washing the residual DNA away from the top of the organic layer, rather than away from the sperm pellet. To date, therefore, all of the methods used by crime laboratories to process vaginal swabs rely on physically separating the residual DNA of the victim from the sperm pellet.

The Erase Sperm Isolation Kit uses an entirely new approach to removing the unwanted residual DNA from the victim, namely by destroying the DNA with a nuclease [20]. The current study demonstrates that use of a nuclease, rather than physical separation methods to remove the residual DNA of the victim, results in the almost complete absence of female DNA as determined by the STR profiles of the male fractions of samples consisting of mock sexual assault cases (female buccal swab cuttings spiked with a known number of sperm), timed post-coital vaginal swabs, and evidence taken from rape victims. In all cases, Erase provided STR profiles that were either equal in quality or superior to those obtained with the standard methods used by the crime laboratories. Surprisingly, Erase worked well the first time it was used in these laboratories. Each person comparing Erase with the standard method was well versed in the standard method and had used it to process many casework samples, while Erase was being used for the first time by these same individuals for this study, suggesting that the Erase protocol is user friendly and robust. In spite of these encouraging results, methodical and direct comparisons of Erase with the standard methods, as outlined above, should be performed by those laboratories interested in this new method before processing casework.

## Abbreviations

PBS: Phosphate-buffered saline; PCR: Polymerase chain reaction; STR: Short tandem repeat.

## Competing interests

AMG is the sole inventor of the intellectual property (US patent application 20080261293) incorporated into the Erase Sperm Isolation Kit and receives royalties based on the sales of this product. All other authors declare that they have no competing interests.

## Authors’ contributions

AMG conceived of the study, prepared the mock samples and wrote the manuscript. AF and JS-G interpreted data, AJ performed the analysis of semen-spiked buccal swab samples and casework samples, MB and MT performed the analysis of semen-spiked buccal swab samples, GS performed validation and interpretation of the data, and NMo performed analysis of semen-spiked buccal swab samples and timed post-coital swabs. VC performed the validation and interpretation of the data. NMa performed analysis of semen-spiked buccal swab samples and validation and interpretation of the data. MM performed analysis of semen-spiked buccal swab samples and the validation and interpretation of the data. All authors read and approved the final manuscript.
